# Tetrodotoxin Poisoning Due to Pufferfish Ingestion in the United Arab Emirates

**DOI:** 10.7759/cureus.33627

**Published:** 2023-01-10

**Authors:** Zahra k Al Dhuhaibat, Talal Zarzour

**Affiliations:** 1 Emergency Medicine, Rashid Hospital, Dubai, ARE; 2 Emergency Medicine, National Health Service, London, GBR

**Keywords:** poisoning of pufferfish, emergency medicine, toxicology, swellfish, neurotoxic, tetrodotoxin

## Abstract

Tetrodotoxin is a potent neurotoxin that is found in the ovaries and liver of pufferfish. This lethal toxin is heat stable and does not destroy by cooking that is why precaution should be taken when eating pufferfish. A 60-year-old male presented to the ED after eating pufferfish complaining of perioral and hand numbness, gait disturbance, and generalized body weakness.

This presentation is due to a tetrodotoxin found in and not limited to the pufferfish he ingested. Despite having pufferfish with his family, he was the sole person to have symptoms because, unlike the other family members, he ate from the liver part of the fish.

The patient was admitted for observation, received supportive care, and underwent multiple investigations that most came to be normal. He improved gradually and was discharged after staying for three nights in the hospital. Symptoms after eating the toxic parts of a pufferfish may progress to paralysis, and respiratory failure, and may lead to death. It is not common to have such fish eaten in the United Arab Emirates due to its limited availability.

## Introduction

Tetrodotoxin (TTX) is one of the most potent neurotoxic poisons found in nature and can occur after consuming it from pufferfish, blowfish, balloon fish, toads, sunfish, porcupine fish, toadfish, globefish, or swellfish [1]. Although pufferfish is considered an Asian delicacy well known as Fugu in Japan, only trained and licensed chefs are allowed to prepare it in restaurants. Much of the fish such as its eyes, blood, liver, and intestines contain the poison. It is not common to have such fish eaten in the United Arab Emirates due to its limited availability [2].

Lagocephalus sceleratus, sometimes referred also as pufferfish or fugu, is a recognized cause of fatal foodborne illness yet is seen as a luxurious dish in Asia. Tetrodotoxin, a powerful toxin found in pufferfishes, is present (TTX). Fatigue, dizziness, paresthesia of the face and limbs, vomiting, and impairment of responses are among the indications that this poison frequently produces [3]. Mortality from respiratory distress and sudden cardiac death happen in cases of extreme poisoning. Along the coastlines of Asian nations, TTX poisoning is frequent.

A very well kind of fish toxicity is pufferfish sickness, which is fatal and is caused by the poison TTX. Reported cases from TTX have been documented in the US, Japan, Taiwan, Hong Kong, Cambodia, and Bangladesh. Solely in Japan, certified pufferfish chefs can typically produce fugu or pufferfish meals. Nevertheless, TTX toxicity has affected 223 Japanese individuals, and between 2002 and 2006, 13 of these individuals passed away [4]. Between 2001 and 2006, 53 individuals in Singapore had TTX toxicity diagnoses, and eight of them passed away. There were three occurrences in Bangladesh in 2008 that affected 141 persons, 17 of whom passed away from respiratory failure. From 1993 to 2006, 23 people and one death were engaged in 10 pufferfish toxicity events in Hong Kong [5]. The major way that TTX impacts the human body is by blocking sodium (Na+) channels in the peripheral and central nervous system, musculoskeletal system, and cardiac muscle cells, which causes a range of clinical characteristics from minor problems to respiratory failure and mortality [6]. The foundation of a prognosis is the people's clinical background, particularly when TTX levels are not available. Through the urine or blood TTX estimate, the prognosis can be verified. Supportive care is the mainstay of pufferfish toxicity treatments. Constant encouragement is the foundation of treatment until the poison is eliminated by urination [7].

Detailed research of a collection of seven pufferfish toxicity instances that presented varying degrees of seriousness was done. While the remaining two manifested with numbing and moderate fatigue, two of the individuals experienced breathing difficulties and needed ventilatory support and breathing assistance. Periorbital tingling was the primary symptom for the majority of them. The typical start of symptoms was 94 minutes long. The poisoning was endured by all participants. A secure and favorable result may be ensured by earlier detection and therapeutic interventions. Despite the rarity of pufferfish toxicity in our everyday practice, emergency doctors should be knowledgeable about the sign and symptoms, and therapy and be prepared to deal with these possibly deadly intoxications [8].

## Case presentation

A 60-year-old male known to have diabetes mellitus type 2 and hyperlipidemia presented with perioral and hand numbness, gait disturbance, and generalized body weakness. He had pufferfish for lunch with his family at his house and tongue numbness started straight away. Then, perioral and hand numbness proceeded. Two hours later, the patient started to be weak and was unable to walk. The patient had visited another hospital in the first seven hours of the pufferfish ingestion and had difficulty breathing five hours post-ingestion where he received treatment for allergic reaction; hydrocortisone, chlorphenamine, ondansetron, and IV fluids were given. During his stay in the other hospital, the patient stated that his vitals were stable including oxygen saturation and blood pressure. Upon arrival at our hospital which was seven hours after the pufferfish ingestion, the patient was placed on a monitor, he remained vitally stable, but symptoms of perioral and hand numbness in addition to the body weakness have persisted with no other complaints. The patient added that the rest of the family had the same fish but only he ate from the liver part of the pufferfish and had symptoms while the rest of the family did not have any symptoms. His cat also had the same part of the fish as him, it vomited then started to have imbalance while walking and got admitted to the veterinarian for just one night.

On examination, the airway was patent with equal bilateral air entry and no added sounds. Neurological examination showed loss of sharp sensation on the fingertips of both hands only, the motor examination of the limbs revealed the power of 5/5 bilateral, he had central body weakness in which he was not able to move his torso at all, and absent reflexes bilaterally. Cranial nerve examination was normal besides tongue weakness. Past pointing on the nose-finger test bilaterally was positive. The rest of the examination was unremarkable. The patient was reviewed by the toxicology team and admitted for supportive care and observation until the resolution of symptoms. The reflexes of the patient came back to normal after 24 hours and his gait gradually improved. Upon discharge, he was mobilizing independently and was having mild dizziness.

Investigations and differential diagnosis 

Electrocardiogram, full blood count, urea and electrolytes, creatinine, liver function tests, lipase, coagulation studies, and cardiac enzymes were all normal. Computerized tomography of the brain was done in the Emergency Department to rule out stroke given his age and co-morbidities which came to be normal. Magnetic resonance imaging of the brain was done on admission because of persistent gait ataxia after 24 hours from consuming the TTX to rule out cerebellar/brainstem pathology which was normal. Neurological conductive studies of upper and lower limbs were done to rule out sensory neuropathy as a cause of gait ataxia and showed mixed sensorimotor polyneuropathy, most likely attributed to his underlying diabetes.

Treatment

The patient received supportive care and physiotherapy during his stay at the hospital without the requirement of intubation and mechanical ventilation.

Outcome and follow-up

The patient started to walk with minimal support and independently after 48 and 72 hours, respectively, from the exposure to the tetrodotoxin. The patient was then discharged home and a follow-up phone call after two weeks revealed the patient to have on-and-off mild dizziness with complete resolution of other symptoms. Also, the cat was doing well since it was discharged from the veterinarian.

## Discussion

TTX is a potent neurotoxin that is found in the ovaries and liver of pufferfish. This lethal toxin is heat stable and does not destroy by cooking which is why precaution should be taken when eating pufferfish [9]. TTX binds to the voltage-gated sodium channels of human muscles and excitable nerve tissues thus inhibiting the sodium ions’ influx and stopping impulse conduction by altering action potential propagation, this results in numbness, nerve paralysis, and immobilization [10]. TTX leads to cardiac, gastric, and CNS-based toxicological symptoms in patients presented with toxicity [11].

There is no specific diagnostic technique available to confirm TTX toxicity. However clinical diagnosis is based on the symptoms and history of the patient. Toxicity symptoms appear within 10 minutes to 6 hours after the ingestion of pufferfish [12]. Toxicity depends upon the concentration of TTX within the body of a patient which is largely caused by ingestion of the liver or ovary part of the fish. TTX can secrete from the body within eight hours and the patient can become symptoms free within 24 hours [13]. Symptoms are classified into four grades depending upon the severity of symptoms. 

Grade I include neuromuscular symptoms of headache, pupil constriction, sweating, and perioral paresthesia with mild gastric symptoms such as abdominal pain, nausea, vomiting, and diarrhea. Grade II toxicity includes early motor paralysis that leads to a lack of coordination. Grade III includes severe neuromuscular symptoms such as ataxia, dysphasia, dysarthria, nerve palsy with tremors, derma-logical symptoms, heart and lung dysfunction, cardiac arrhythmias, and hypotension. Grade IV is considered severe pufferfish toxicity (TTX) with respiratory paralysis, delirium, arrhythmia, and severe hypotension [14]. 

There is no specific antidote available for TTX poisoning and only supportive therapy is given [6]. Gastric lavage, dialysis, and early endotracheal intubation are recommended with the additional use of Neostigmine to treat acute respiratory failure caused by TTX accumulation in the body [15]. 

Alhatali et al. performed an observational study to report various cases of TTX poisoning in Oman. The study documented that five patients with TTX poisoning are presented in the hospital by intake of internal organs of fried local pufferfish from the coast of Oman. Clinical manifestations of patients include upper and lower limbs weakness, paralysis, generalized paresthesia, dyspnea, bradycardia, hypotension, and Coma. Four patients recovered completely except one who had developed subarachnoid hemorrhage. Supportive treatment was given with neostigmine and intermittent dialysis. The patient underwent complete recovery within 24 hours [16].

Similarly, a 60-year-old patient reported to the hospital with numbness around his mouth and presented with a history of eating 50g pufferfish two hours before the appearance of symptoms. Vital signs were stable with no other profound symptoms. The patient was given gastric lavage immediately. The patient suddenly became cyanotic with loss of consciousness; he had a cardiac arrest and cardiopulmonary resuscitation had been started immediately. After the restoration of symptoms, the patient was shifted to the Intensive care unit (ICU) with the complications of sinus tachycardia. His serum concentration of TTX was noted as 348ng/mL. He was given mechanical ventilation with all comprehensive treatment. Spontaneous breathing was not present during the ICU stay, no pupillary reflex was present, and the patient was in Coma and eventually died of respiratory failure [12].

Due to the well-known severity of toxicity of pufferfish, many countries have banned its import, consumption, and trade worldwide [17]. In Asia TTX poisoning is one of the most frequently historically known fish poisoning, many countries have legislation while Japan has more articulate legislation regarding its cooking and import of pufferfish in a restaurant by certified Chefs, only on special occasions [18]. FDA has restricted its import in the USA. Restrictions are also present in China, Vietnam, Thailand, and Taiwan [19].

The prognosis of TTX poisoning is poor and the mortality rate is about 13.5% to 30%. The best way is to take precautionary measures, avoid eating its ovary and liver part and generate awareness about its toxic profile. Soaking for a long duration and cooking for many hours at a high temperature can lessen its toxicity [20]. 

There is limited literature available as evidence against TTX toxicity and its management. Further research studies are required for its treatment and prevention.

## Conclusions

TTX is a serious neurotoxin that can lead to respiratory arrest and death. Environmental health is a vital part of medicine for both public to know the dangers of certain poisons found in nature like the pufferfish and for healthcare providers to treat them. We study the case of a 63-year-old man with TTX poisoning. The man had a history of diabetes mellitus type 2 and hyperlipidemia. The patient suffered from numbness, little respiratory problems, and an inability to move. However, after receiving supportive care from the hospital his mobility came back after 24 hours and his medical examination also showed positive results. Hence, we conclude that timely diagnosis of TTX poisoning and management saved our patient from any deadly results.

## References

[1] Kotipoyina H, Kong E, Warrington S (2020). Tetrodotoxin Toxicity.

[2] Kheifets J, Rozhavsky B, Girsh Solomonovich Z, Marianna R, Soroksky A (2012). Severe tetrodotoxin poisoning after consumption of Lagocephalus sceleratus (pufferfish, fugu) fished in Mediterranean Sea, treated with cholinesterase inhibitor. Case Rep Crit Care.

[3] Al Homsi A (2022). A case report of puffer fish poisoning from United Arab Emirates (PREPRINT). Oman Med J.

[4] Noguchi T, Onuki K, Arakawa O (2011). Tetrodotoxin poisoning due to pufferfish and gastropods, and their intoxication mechanism. Int Scholarly Res Notices Toxicol.

[5] Lago J, Rodríguez LP, Blanco L, Vieites JM, Cabado AG (2015). Tetrodotoxin, an extremely potent marine neurotoxin: distribution, toxicity, origin and therapeutical uses. Mar Drugs.

[6] Torda TA, Sinclair E, Ulyatt DB (1973). Puffer fish (tetrodotoxin) poisoning: clinical record and suggested management. Med J Aust.

[7] Ahasan HA, Al Mamun A, Rasul CH, Roy PK (2005). Puffer fish poisoning (tetrodotoxin) in Bangladesh: clinical profile and role of anticholinesterase drugs. Trop Doct.

[8] Wan C., Tsui S, Tong H (2007). A case series of puffer fish poisoning. Hong Kong J Emerg Med.

[9] Kiernan MC, Isbister GK, Lin CS, Burke D, Bostock H (2005). Acute tetrodotoxin-induced neurotoxicity after ingestion of puffer fish. Ann Neurol.

[10] Grattan LM, Kilmon KA, Fiore A (2011). Seafood intoxication. Foodborne Infections and Intoxications.

[11] Madejska A, Michalski M, Osek J (2019). Marine tetrodotoxin as a risk for human health. J Vet Res.

[12] Zhu S (2021). A fatal case of severe pufferfish poisoning: a case report. Medicine: Case Reports and Study Protocols.

[13] Arakawa O (2010). Toxins of pufferfish that cause human intoxications. Coastal Environmental Ecosystem Issues East China Sea.

[14] Chowdhury FR, Nazmul Ahasan HA, Mamunur Rashid AK, Al Mamun A, Khaliduzzaman SM (2007). Tetrodotoxin poisoning: a clinical analysis, role of neostigmine and short-term outcome of 53 cases. Singapore Med J.

[15] Liu SH, Tseng CY, Lin CC (2015). Is neostigmine effective in severe pufferfish-associated tetrodotoxin poisoning?. Clin Toxicol (Phila).

[16] Alhatali B, Al Lawatia S, Khamis F (2022). A cluster of tetrodotoxin poisoning in Oman. Clin Toxicol (Phila).

[17] Nagashima Y (2016). Dangerous marine animals. Food poisonings due to marine biotoxins of fishes. Jpn J Toxicol.

[18] Noguchi T, Arakawa O (2008). Tetrodotoxin--distribution and accumulation in aquatic organisms, and cases of human intoxication. Mar Drugs.

[19] Cong NH, Tuan le TQ (2006). Electrodiagnosis in puffer fish poisoning--a case report. Electromyogr Clin Neurophysiol.

[20] Noguchi T, Ebesu JS (2001). Puffer poisoning: epidemiology and treatment. J Toxicol: Toxin Rev.

